# Associations between radiologist-defined semantic and automatically computed radiomic features in non-small cell lung cancer

**DOI:** 10.1038/s41598-017-02425-5

**Published:** 2017-06-14

**Authors:** Stephen S. F. Yip, Ying Liu, Chintan Parmar, Qian Li, Shichang Liu, Fangyuan Qu, Zhaoxiang Ye, Robert J. Gillies, Hugo J. W. L. Aerts

**Affiliations:** 1 Department of Radiation Oncology, Dana-Farber Cancer Institute, Brigham and Women’s Hospital, and Harvard Medical School, Boston, MA 02115 USA; 2 Department of Radiology, Tianjin Medical University Cancer Institute and Hospital, National Clinical Research Center for Cancer, Key Laboratory of Cancer Prevention and Therapy, Tianjin; Tianjin’s Clinical Research Center for Cancer, Tianjin, PR China; 3 Department of Cancer Imaging and Metabolism, H. Lee Moffitt Cancer Center and Research Institute, Tampa, FL USA; 4 Department of Radiology, H. Lee Moffitt Cancer Center and Research Institute, Tampa, FL USA; 5 Department of Radiology, Brigham and Women’s Hospital and Harvard Medical School, Boston, MA 02115 USA

## Abstract

Tumor phenotypes captured in computed tomography (CT) images can be described qualitatively and quantitatively using radiologist-defined “semantic” and computer-derived “radiomic” features, respectively. While both types of features have shown to be promising predictors of prognosis, the association between these groups of features remains unclear. We investigated the associations between semantic and radiomic features in CT images of 258 non-small cell lung adenocarcinomas. The tumor imaging phenotypes were described using 9 qualitative semantic features that were scored by radiologists, and 57 quantitative radiomic features that were automatically calculated using mathematical algorithms. Of the 9 semantic features, 3 were rated on a binary scale (cavitation, air bronchogram, and calcification) and 6 were rated on a categorical scale (texture, border definition, contour, lobulation, spiculation, and concavity). 32–41 radiomic features were associated with the binary semantic features (AUC = 0.56–0.76). The relationship between all radiomic features and the categorical semantic features ranged from weak to moderate (|Spearmen’s correlation| = 0.002–0.65). There are associations between semantic and radiomic features, however the associations were not strong despite being significant. Our results indicate that radiomic features may capture distinct tumor phenotypes that fail to be perceived by naked eye that semantic features do not describe and vice versa.

## Introduction

Medical imaging is an indispensable clinical tool for cancer diagnosis, staging, and therapeutic assessment. In particular, computed tomography (CT) is the most widely used imaging modality and is the standard of care for lung cancer management^1, 2^. Lung cancer is the deadliest cancer type with a 5-year overall survival rate of only about 15% and affects over 1.5 million patients worldwide^3^. Several studies have indicated that the identification of unique characteristics of individual lung tumors may provide clinicians with crucial information to personalize treatments for patients^4, 5^. These unique characteristics can be qualitative CT-based descriptors, termed semantic features, that describe a tumor’s shape and internal structure that are scored by radiologists to characterize lung lesions^5–8^. Semantic features have been shown to predict prognosis^9–13^, therapeutic response^14, 15^, and genetic mutations^16–18^ in patients with lung cancer. For example, a tumor with cavitation has been shown to be an indicator of high aggressiveness and poor prognosis, based on the rationale that fast growing tumors may exceed the growth of their blood supply resulting in air-filled cavities arising from central necrosis^9^. Semantic features are considered qualitative since they are scored according to the visual assessment of radiologists, which limits the extent of the tumor description to what is observable by the eye.

The quantitative nature of CT allows numerous imaging features to be defined using advanced mathematical algorithms to describe tumor shape, image intensity distribution, and the relationship between image voxels in great detail that may fail to be perceived by the naked eye of physicians–even experienced radiologists^19–22^. Radiomics is a field that extracts these imaging features to quantitatively characterize the tumor phenotype with high-throughput^23, 24^. Many groups have reported that radiomic features may predict overall survival^19, 25–27^, distant metastasis^28–30^, treatment response^31–33^, and somatic mutations^34–36^ in lung cancer patients, as well as other malignancies.

While both semantic and radiomic features have been investigated for their promise in characterizing tumors for personalized therapy, the associations between the two feature types has yet to be investigated. Understanding the association between these two types of features may shed light on their complementary nature in outcome and genetic prediction. Furthermore, radiomic features are known as agnostic features as they are difficult to intuitively interpret or describe^37^, however, understanding their association with semantic features may help interpret some of the radiomic features, based on their highly correlated semantic counterparts. In this study, we investigated the relationship between various semantic and radiomic features in 258 patients with lung adenocarcinoma.

## Results

Our study cohort consisted of 183 early stage (Stage I and II) and 75 advanced stage (stage III and IV) patients with non-small cell lung adenocarcinoma (Table 1). This study investigated the association between 9 semantic and 57 radiomic features. Although 296 radiomic features were initially extracted from CT images, only 57 features (10 unfiltered and 47 filtered features) with |ρ| ≤ 0.85 were included to evaluate their relationship with semantic features. Cavities, tube-like or branched air structures (air bronchogram) were found in over 50% of the tumors, while only 11% of the tumors were calcified (Table 1). The majority of the tumors had a solid texture with somewhat irregular contours and slight concavity (Table 1).

**Table 1:** Patient characteristics. Distribution of patient tumor characteristics and radiologists’ scoring for semantic features.

**Number of Patients**	Total
	258
**Sex**	
Male/Female	146 (57%)/112 (43%)
**Median age** (year)	59 (range 30–81)
**Smoking history**	
Current or Former/Never	117 (45%)/141 (55%)
**Clinical stage**	
I/II/III/IV	160 (62%)/23 (9%)/66 (26%)/9 (3%)
**Histology subtype**	
Minimally invasive adenocarcinoma	3 (1%)
Acinar predominant	109 (42%)
Lepidic predominant	60 (23%)
Papillary predominant	20 (8%)
Micropapillary predominant	12 (5%)
Solid predominant	49 (19%)
Variants of invasive adenocarcinomas	5 (2%)
**Tumor grade**	
Low/Intermediate/High	3 (1%)/189 (73%)/66 (26%)
**CT Scanners**	
Siemens	
Somatom Sensation 64	30 (12%)
GE scanner	
Lightspeed 16/Discovery CT750 HD	35 (14%)/193(75%)
**Binary semantic features**	
Cavitation (score: 0/1)	106 (41%)/152 (59%)
Air Bronchogram (score: 0/1)	116 (45%)/142 (55%)
Calcification (score: 0/1)	229 (89%)/29(11%)
**Categorical Semantic features**	
Texture (score: 1/2/3)	6 (2%)/68 (26%)/184 (71%)
Border definition (score: 1/2/3)	13 (5%)/178 (69%)/67 (26%)
Contour (score: 1/2/3/4)	17 (7%)/26 (10%)/166 (64%)/49 (19%)
Lobulation (score: 1/2/3/4)	10 (4%)/115 (45%)/102 (40%)/31 (12%)
Spiculation (score: 1/2/3)	63 (24%)/85 (33%)/110 (43%)
Concavity (score: 1/2/3)	9 (4%) /156 (61%)/93 (36%)

### Binary semantic features

**Figure 1:**
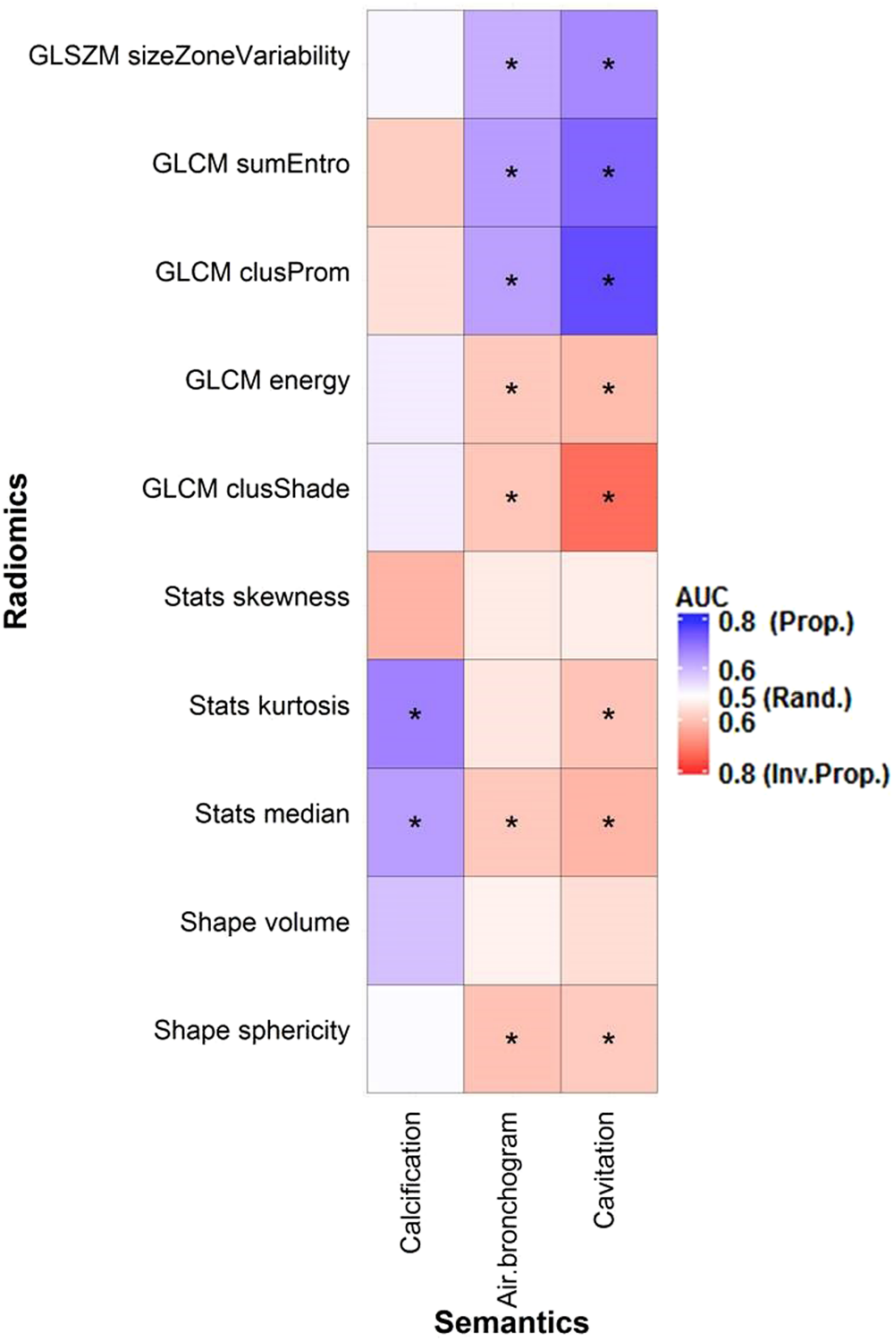
Association between the binary semantic and unfiltered radiomic features assessed with the area under the ROC curve (AUC). *Indicates a significant association (q-value ≤ 0.05). “Rand.” = random association (AUC = 0.50). “Prop.” and “Inv. Prop.” indicate direct and inverse proportionality, respectively.

**Figure 2:**
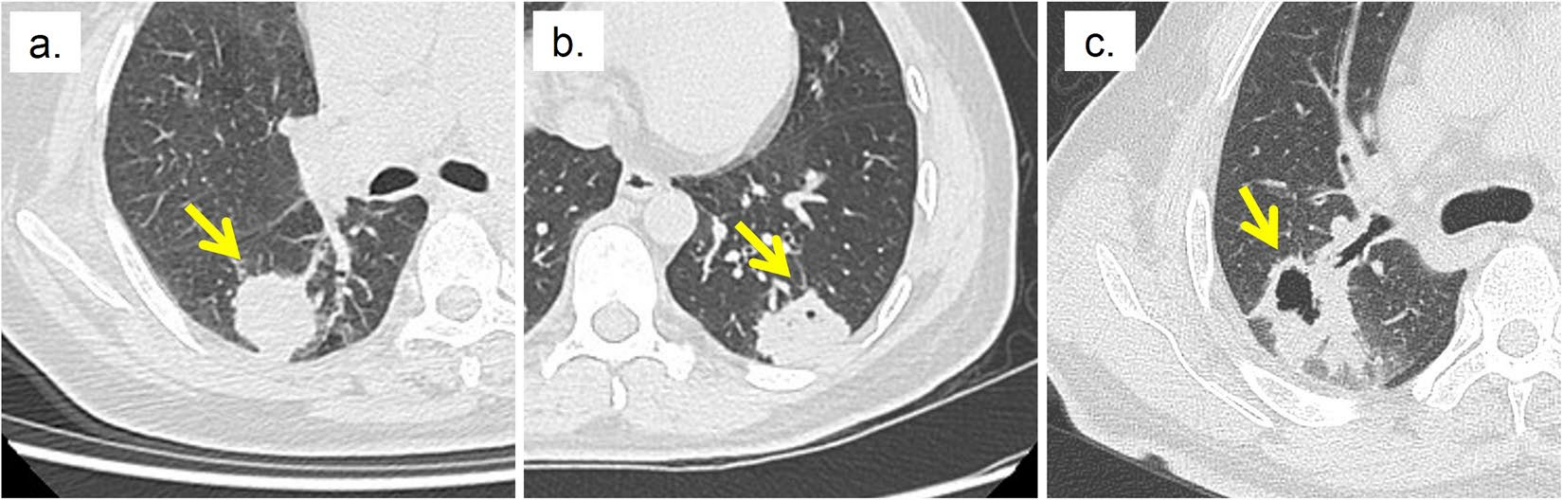
Tumors with and without cavitation. (**a**) Tumor without cavitation (**b**) Tumor with minor Cavitation (**c**) Tumor with major Cavitation. The arrow indicates the location of the tumor.

**Figure 3:**
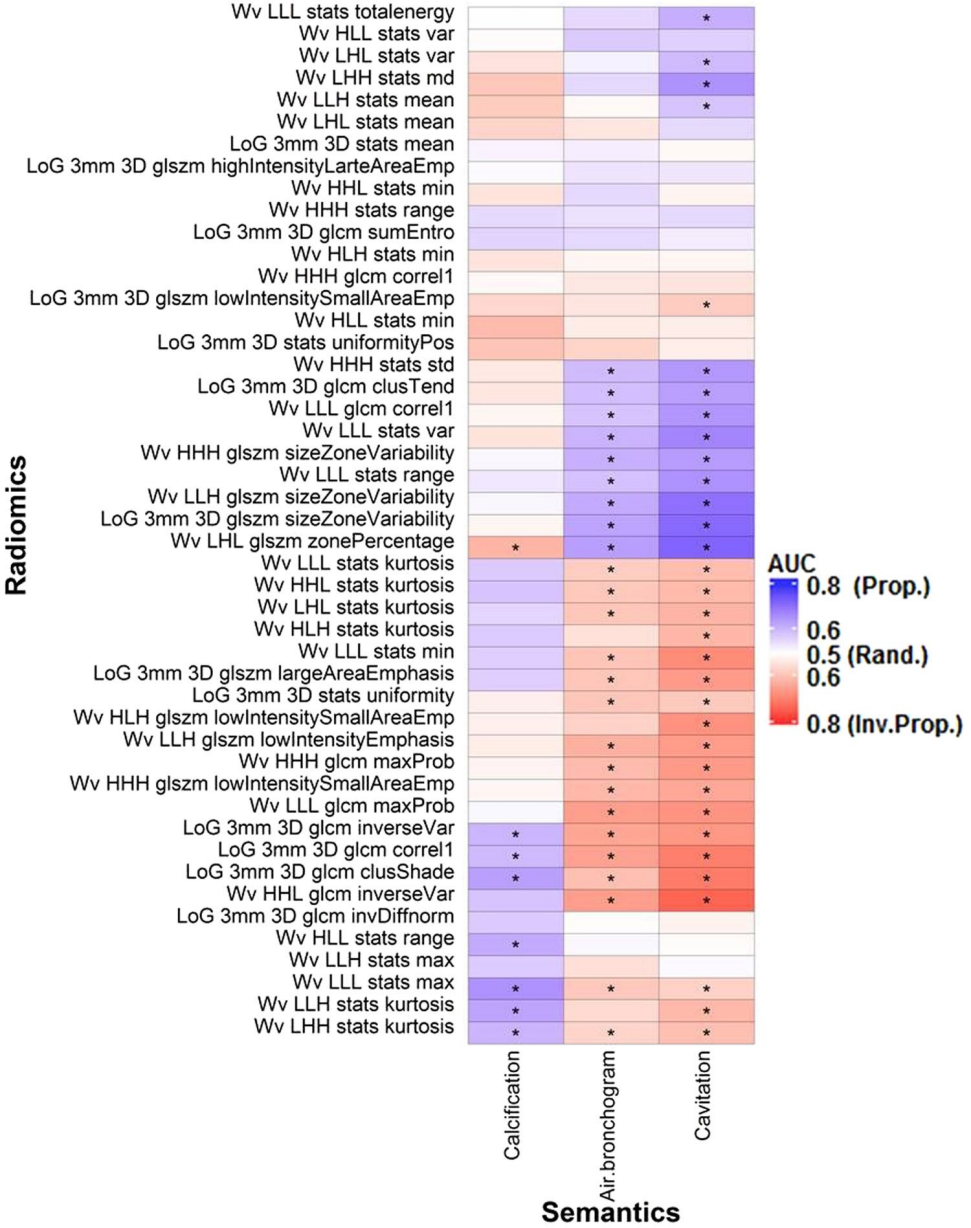
Associations between the binary semantic and unfiltered radiomic features assessed with the area under the ROC curve (AUC). *Indicates a significant association (q-value ≤ 0.05). “Rand.” = random association (AUC = 0.50). “Prop.” And “Inv. Prop.” indicate direct and inverse proportionality, respectively. Wv = Wavelet. LoG = Laplacian of Gaussian.

The area under the receiver operating characteristic curve (AUC) was used to quantify the association between binary semantic and radiomic features. Tumors with cavitation, tube-like or air branched structures (air bronchogram) were associated with low values of shape-based sphericity and had lower values for features that described homogeneity (e.g. gray level co-occurrence matrix derived (GLCM) energy), but greater values for features that described heterogeneity (e.g. gray level size zone matrix derived (GLSZM) size-zone-variability) (Fig. 1). For instance, as observed in Fig. 2, tumors with cavitation were less spherical and more heterogeneous than those without cavitation. 41, 32, and 10 radiomic features were significantly related to cavitation (AUC_prop_ = 0.59–0.76, AUC_inv-prop_ = 0.56–0.75), air bronchogram (AUC_prop_ = 0.59–0.64, AUC_inv-prop_ = 0.57–0.66), and calcification (AUC_prop_ = 0.60–0.68, AUC_inv-prop_ = 0.62), respectively. In particular, GLCM-Cluster Prominence (AUC_prop_ = 0.76), Wavelet high-high-low pass filtered (HHL) GLCM-inverse Variability (AUC_inv-prop_ = 0.66), and Kurtosis (AUC_prop_ = 0.68) were most strongly associated with cavitation, air bronchogram, and calcification, respectively (Figs 1 and 3). Supplementary Table S2 and S3 show the AUC and q-values for all the features. However, tumor volume, statistic-based skewness, 5 Laplacian of Gaussian (LoG) and 8 wavelet filtered features were not significantly associated with any of the binary features (AUC_prop_ = 0.51–0.59, AUC_inv-prop_ = 0.50–0.63; q-value ≥ 0.70).

### Categorical semantic features

**Figure 4:**
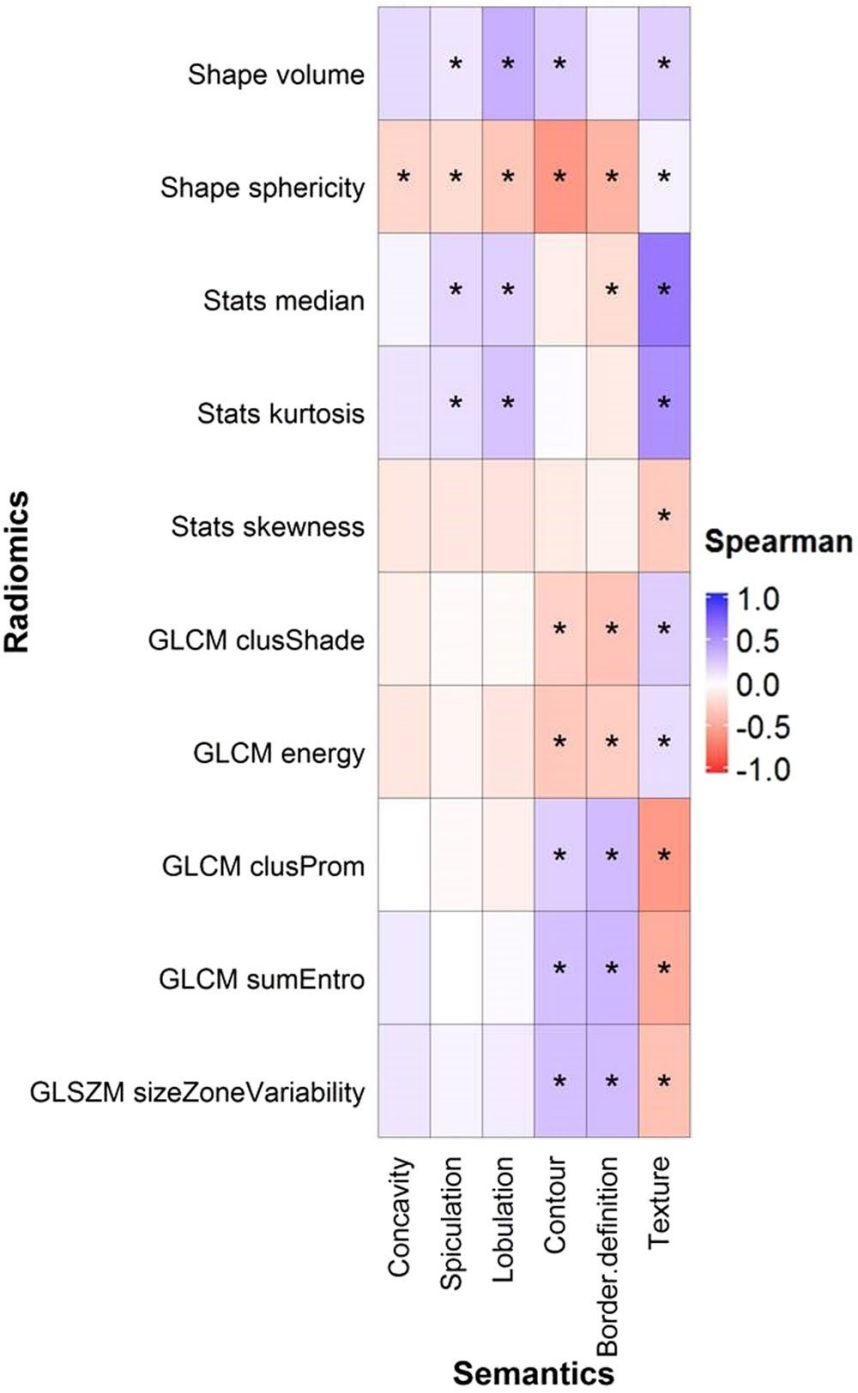
Association between the six categorical semantic and ten unfiltered radiomic features assessed with Spearman coefficient correlation. *Indicates that the association was significant (q-value ≤ 0.05).

**Figure 5:**
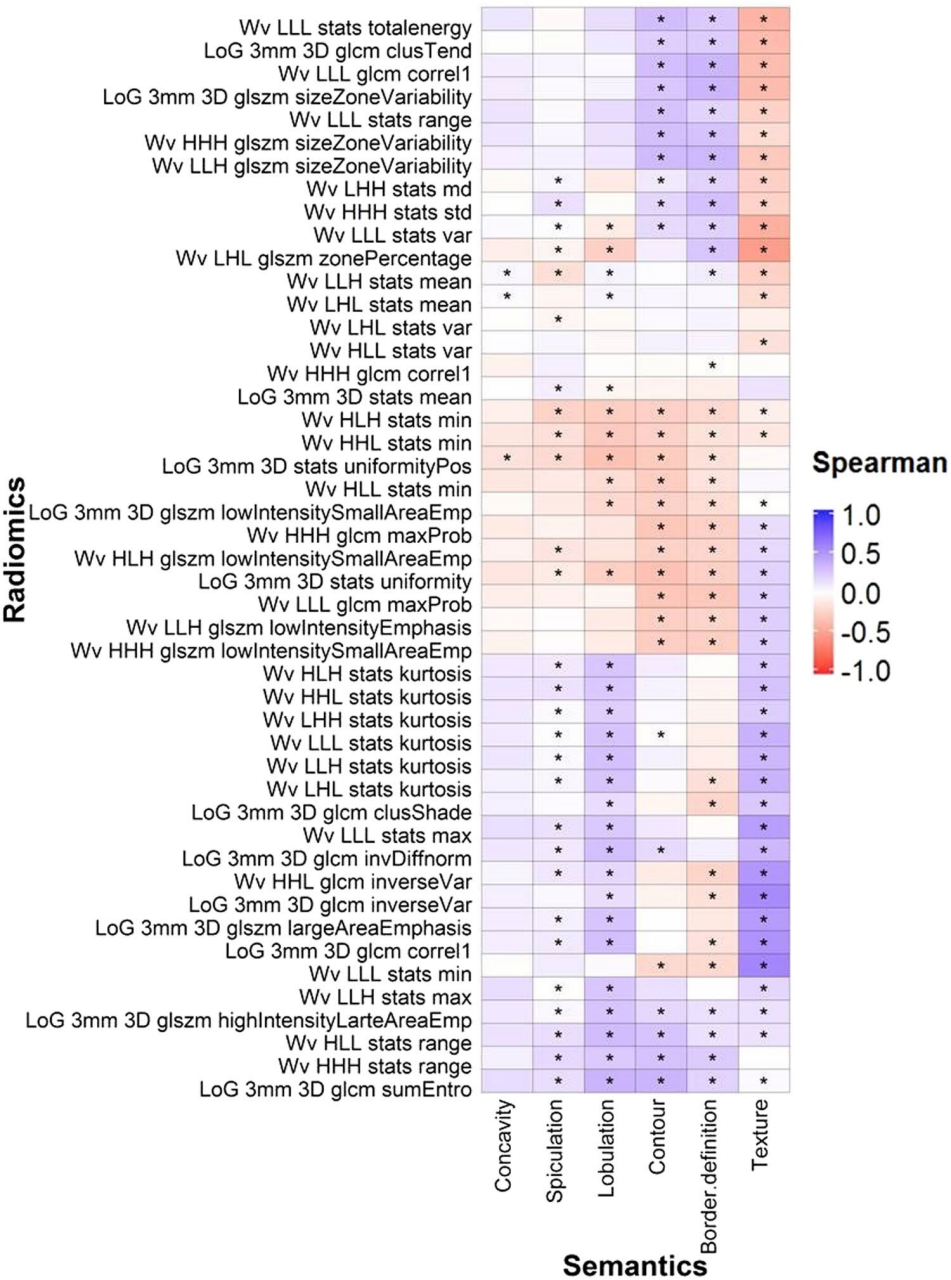
Associations between the categorical semantic and unfiltered radiomic features assessed with Spearman coefficient correlation. *Indicates a statistically significant association (q-value ≤ 0.05). Wv = Wavelet. LoG = Laplacian of Gaussian.

Radiomic features were also associated with categorical semantic features. Over 30 radiomic features were significantly related to texture (51 radiomic features), border definition (41 radiomic features), contour (35 radiomic features), lobulation (33 radiomic features), and spiculation (32 radiomic features) as evaluated with the Kruskal Wallis test (Figs 4 and 5). Only four radiomic features were found to have a significantly association with concavity (Figs 4 and 5).

**Figure 6:**
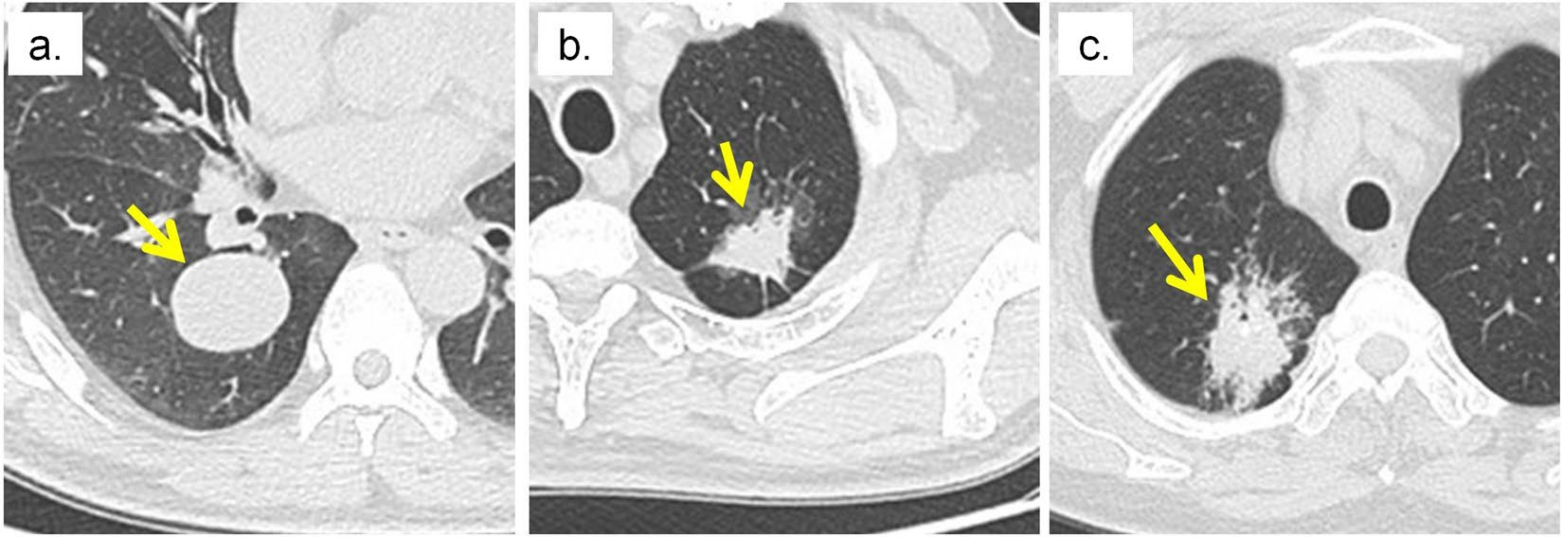
Tumors with different border definitions. (**a**) Tumor with a well-defined border (score = 1). (**b**) Tumor with neither a well- or poorly-defined border (score = 2). (**c**) Tumor with a poorly-defined border (score = 3). The arrow indicates the location of the tumor.

Tumors with non-solid or poorly defined boundaries generally had higher values in features that quantified heterogeneity (e.g. GLSZM size zone variability) (Fig. 4). Tumors with strong lobulation and spiculation, deep concavity, or poorly-defined borders were inversely correlated with shaped-based sphericity (Figs 4 and 6). For example, Fig. 6 shows that tumors with well-defined borders are more spherical and homogeneous than tumors with irregular and poorly-defined borders. The absolute correlations (|ρ|) between these radiomic features and texture, border definition, contour, lobulation, spiculation, and concavity were 0.002–0.65 (median = 0.26), 0.01–0.42 (median = 0.24), 0.01–0.57 (median = 0.28), 0.05–0.38 (median = 0.27), 0.003–0.25 (median = 0.11), and 0.02–0.23 (median = 0.10), respectively (Fig. 4). Shape-based sphericity was significantly associated with all categorical features and was most correlated with border definition, contour, and concavity. The median value of the tumor image intensity (Hounsfield Unit), Wavelet HLH statistics-based minimum, and tumor volume was most associated with texture, spiculation, and lobulation, respectively. Supplementary Table S4 and S5 show the ρ and Kruskal-Wallis test q-values for all the features.

## Discussion

Semantic features are qualitative imaging features that are defined by experienced radiologists and have shown to be promising predictors of the aggressiveness of lung adenocarcinoma^5, 24, 37^. Radiomic features are automatically and quantitatively extracted from CT images using advanced mathematical algorithms that have also shown great potential to predict clinical outcomes and describe tumor heterogeneity^23^. Assessing the relationship between both types of features may help understand their complementary nature for outcome prediction and may allow a better and more intuitive interpretation of radiomic features. We investigated the relationship between 9 semantic and 57 radiomic features in lung adenocarcinoma patients.

When describing tumor characteristics, global qualitative features of tumors (e.g. border, roundness, and interior texture) are most noticeable to radiologists. However, radiomic features are based on mathematical algorithms that describe tumor phenotypes that may not be noticeable to radiologists. Since both types of features aim to describe the tumor appearance captured on CT images, it is not surprising that all semantic features were associated with at least four radiomic features (Figs 1, 3, 4 and 5). In particular, shape-based sphericity was significantly related to all semantic features, except calcification (Figs 1 and 4). Sphericity quantifies the roundness of a tumor and is considered as a dominate feature of the tumor which may relate to the semantic features which also have some dependence on roundness.

Round tumors with a smooth border may tend to be more indolent. Lobulation and spiculation describe the undulating patterns and spikes on the tumor borders. Shape-based sphericity was negatively correlated with lobulation and spiculation, thus indicating that round tumors have fewer undulations and spikes. Indeed, tumors with no spiculation, no lobulation are less likely to be associated with local and distant metastasis and poor survival^7, 12, 38, 39^. Furthermore, the surrounding bronchus and blood vessels may hamper isotropic enlargement of the tumor leading to the “notch” appearance (or concave cuts) in its boundary. We also observed that irregular and non-spherical tumors often had higher concavity (Fig. 6 and Supplementary Figure S2). Tumors with high concavity are often an indicator of poorly differentiated adenocarcinoma and outcomes^40–42^. Our observations were thus consistent with the previous studies that tumors with irregular and non-spherical shape are more aggressive, and thus are poor prognostic indicators^12, 43, 44^.

Tumors with a high median intensity were more likely to be calcified or solid. Single to multiple calcium “spots” can be observed on the calcified tumors. These spots were usually small and did not distort or contribute to the overall structure of the tumor (Supplemental Figure S1). This may explain why the relationship between shape-based Sphericity and calcification was not significant. Studies have reported that calcium layers generally have higher image intensity than tumor tissues^45–47^. We also found in this study that calcified tumors were significantly associated with higher median image intensity. Ground glass opacity (GGO) lesions refer to hazy regions with slightly increased CT attenuation in the lung without obscuring the visibility of normal lung parenchyma, airways, and vessels^48^ (Supplemental Figure S2). While partly solid tumors only partially obscure the bronchial and vascular structures, solid tumors completely obscured these structures (Supplemental Figure S2). Due to the hazy appearance of GGO (non-solid) tumors, their median image intensity was less than partly solid and solid tumors (Fig. 4). In addition, since GGO and partly solid tumors do not completely obscure the bronchi and vessels, they often appear to be more heterogeneous than solid tumors. Non- or partly solid tumors often had lower values in homogenous features (e.g. GLCM-Energy), but higher values in heterogeneous features (e.g. GLSZM-Size Zone Variability) than solid tumors (Fig. 4). Furthermore, GGO and partly solid tumors can be further described by radiologists as well-defined/coarse interface, the proportion of consolidation, or bronchus cut-off, etc^49, 50^. However, our radiologists only classified the textures of tumors into GGO, partly solid, and solid lesions. In the future, it would be interesting to investigate the relationship between these sub-semantic categories and radiomic features, specific to GGO or partly solid tumors.

Radiomic features that quantify the spatial relationship between image voxels (textural features) may be useful to measure the tumor cavitation and air bronochogram. Tumor cavitation and air bronchogram were significantly and moderately associated with all textual radiomic features (Fig. 1). GLCM-Cluster Shade and GLCM-Cluster Prominence emulate human perception and measure asymmetry and intensity variation within the tumors^51^. Textural features, for instance GLCM-Energy and GLSZM-Size Zone Variability, measure the degree of spatial intensity variability in a tumor^52^. Cavitation is often observed in rapidly growing tumors as they can outgrow the blood supply resulting in air-filled cavities^8, 53, 54^. Tumors with air bronchogram contain tube-like structures and are highly metastatic^40, 44^. Cavities and tube-like structures give tumors heterogeneous appearance (Fig. 2).

The binary and categorical scales employed to rate semantic features may be insufficient to describe subtle tumor characteristics. However, radiomic features have values on a continuous scale which can provide greater detail for changes in tumor characteristics. Despite the significant relationships between semantic and radiomic features, such relationships only ranged from weak to moderate. For example, although the contour semantic feature and the shape-based sphericity radiomic feature both measure the roundness of the tumor, they were only moderately correlated (ρ = −0.57, Fig. 4). Notably, shape-based Sphericity is a continuous feature whereas contour was rated on a categorical scale. Another example is cavitation. As observed in Fig. 2, tumors can exhibit various degrees of cavitation. However, tumors are only rated either with or without cavitation and make no differentiation between high or low degrees of cavitation.

Furthermore, while GLCM-Cluster Prominence‒a intensity variability measure‒can be used to detect small intensity differences between image voxels^51^, radiologists may fail to identify such variations. It is assumed that radiomic features are able to capture tumor characteristics fail to be identified by radiologists^22, 23^. The weak to moderate relationship between semantic and radiomic features may thus be due to the fact that radiologists cannot detect the subtle change in tumors using the categorical scales. Additionally, it has been reported that radiologists may overlook salient features on CT and chest X-ray, even around regions with lung abnormalities^55, 56^. Since objective radiomic features are continuous, they may have advantage over subjective semantic features in identifying imaging phenotypes, which may fail to be perceived by the naked eye, for tumor characterization^20, 57^.

Thus far, our discussion has focused on unfiltered features, such as shape-based sphericity and GLCM-based energy, because filtered features are more difficult to interpret. A LoG filter involves applying the Gaussian filter to an image to remove random noise while a Laplacian filter is employed to enhance strong features on the image. A wavelet transformation decomposes the low (coarse feature) and high (fine feature) frequency regions of an image^58, 59^. Both Coroller *et al*.^28^ and Huynh *et al*.^29^ found that the Wavelet LLH stats range was significantly predictive of distant metastasis in lung cancer in both their datasets. Wavelet LLH stats range and Wavelet LLH stats max were highly correlated in our dataset (ρ = 0.95, results not shown). Wavelet LLH stats max may also be correlated to lung tumor metastatic potential. It is not surprising that Wavelet LLH stats max was significantly associated with spiculation and lobulation (Fig. 4) since tumors with coarse spiculation and lobulation are likely to be invasive.

In our study, all of the patients had non-contrast-enhanced CT images. A recent study by He *et al*.^60^ investigated the impact of various CT acquisition parameters (i.e. contrast-enhancement, slice thickness, and convolution kernel) on the diagnostic performance of radiomic features in pulmonary nodules. Although contrast agents may obscure imaging features that reflect the underlying intra-tumoral heterogeneity, features computed with different types of CT images were both predictive of the nodule malignant status with <5% difference in the AUCs (i.e. AUC_non-contrast_ = 0.86 vs AUC_contrast_ = 0.82 in the training and AUC_non-contrast_ = 0.75 and AUC_contrast_ = 0.74 in the validation cohort). Therefore, the association between semantic and radiomic features based on the contrast enhanced CT should still range from weak to moderate as observed in our current study.

## Conclusion

A number of radiomic features were significantly associated with semantic features. However, the associations only ranged from weak to moderate, suggesting that both types of feature can potentially provide information that captures tumor phenotypes differently. As both semantic and radiomic features have shown promise in identifying aggressive tumors, their complementary roles in outcome prediction needs to be further investigated.

## Materials and Methods

### Patient and CT imaging

In this retrospective study, all experimental and imaging protocols were approved by an Institutional Review Board at the Tianjin Medical University Cancer Institute and Hospital (Tianjin, PR China) and informed consent was waived for all the participants. All methods were also performed in accordance with relevant guidelines and regulations. The cohort consisted of 258 Asian patients with pathological confirmation of lung adenocarcinoma either by surgical specimens or biopsy sample between November 2012 and March 2014. Table 1 shows the patient characteristics.

Chest CT images were acquired on one of the three multiple detector CT scanners: Somatom Senation 64 (Siemens AG, Erlangen, Germany), Lightspeed 16, or Discovery CT750HD (GE Healthcare, Waukesha, WI) prior to any treatments. The CT images acquired on the 64-detector Siemens scanner were scanned with a tube voltage of 120 kVp, automatically adjusted current, pitch of 0.969, and were reconstructed with a 1.5 mm slice thickness. The image acquisition parameters for both GE scanners were 120 kVp and 150–200 mA with a pitch of 0.969. The reconstructed images acquired on the GE scanners had a slice thickness of 1.25 mm.

### Semantic features

Three experienced thoracic radiologists (Y.L., F.Q., and S.L.) independently reviewed all CT images and assigned scores to each tumor for nine semantic imaging features. All radiologists were blinded to the scores assigned by the other radiologists. The score that was chosen by the majority of the radiologists was recorded for that semantic feature. If none of the radiologists had the same score, they reviewed the CT images together and any discrepancies were resolved by discussion until consensus was reached. Three semantic features (cavitation, air bronchogram, and calcification) were scored on a binary scale and rated as having the presence (score = 1) or absence (score = 0) of characteristic. The following semantic features were scored on categorical scales, ranging from 1 to 4: texture, border definition, contour, lobulation, spiculation, and concavity. The semantic features that were scored on the binary or categorical scales are hereafter referred to as binary or categorical features, respectively. The definitions and scoring scale of each semantic feature is shown in Table 2. Visual examples of tumors with different semantic features are shown in the Supplementary Information (Supplementary Figures S1 and S2 in Supplemental).

**Table 2:** Definition of the CT-based semantic features for lung tumor. Visual examples of tumors with different semantic features are shown in the supplemental materials.

Semantic feature type	Semantic feature	Definition	Scoring
**Binary Features**			
	Cavitation	Gas-filled space (cavity) within the tumor due to central necrosis	1 = presence, 0 = absence
	Air Bronchogram	Tubular line or branched air structure within the tumor	1 = presence, 0 = absence
	Calcification	Display layer(s) of calcium in any patterns	1 = presence, 0 = absence
**Categorical Features**			
	Texture	Non-solid/GGO, part-solid or solid tumor	1 = non-solid/GGO; 2 = part-solid; 3 = solid
	Border definition	Appearance of the edge of the tumor	1 = well defined; 2 = tumor border is neither poor nor well defined; 3, poorly defined
	Contour	Roundness of the tumor	1 = round; 2 = oval; 3 = somewhat irregular; 4 = irregular
	Lobulation	Tumor with undulating border	1 = not lobulated; 2 to 4 = lobulated tumor with increasing degree
	Spiculation	Tumor with spikes its edge	1 = no spiculation; 2 = fine spiculation; 3 = coarse spiculation
	Concavity	Notches (or concave cut) on the tumor surface	1 = no concavity; 2 = slight concavity; 3 = deep concavity

### Tumor volume segmentation and radiomic feature extraction

Tumor volume segmentation were performed on the Definiens Developer XD^©^ (Munich, Germany) imaging platform. Tumor volumes were segmented using the single-click ensemble segmentation (SCES) algorithm^61^ and a region growing algorithm^62^. Briefly, two radiologists (Y.L. and Q.L.) identified tumor regions for automatic seed point generation using SCES. A region growing algorithm was then performed on each seed point to create the tumor volume. The segmented tumor volumes were then reviewed slice-by-slice and manually adjusted by the radiologists (Y.L. and Q.L.). A detailed description of the tumor segmentation process can be found in our previous studies^61, 63, 64^. All tumor segmentations were performed on the chest CT images based on the lung window settings.

All radiomic features were computed using an in-house software based on MATLAB (The Mathworks Inc, Natick, MA, U.S.A.). Within the segmented tumor volumes, 13 shape features, 12 statistics features, and 23 textural features were extracted from the CT images. The textural features included 17 gray level co-occurrence matrix (GLCM), 1 gray level size zone matrix (GLSZM), and 5 run length gray level (RLGL) features.

Laplacian of Gaussian (LoG) and wavelet filters are often applied to medical images prior to textural feature extraction^19, 28, 65^. LoG and wavelet filters were applied to the CT images and an additional 247 radiomic features were extracted. In total, 294 radiomic features (47 unfiltered and 247 filtered features) were computed.

### Radiomic feature selection

Spearman’s correlation coefficient (ρ) was used to assess the correlation between all radiomic features. Feature pairs with |ρ| ≥ 0.85 were considered to be strongly correlated and likely to provide redundant information about the tumor phenotype. In these strongly correlated pairs, feature with the highest average |ρ| was excluded. After the exclusion, 10 unfiltered radiomic features (two shape (volume and sphericity), three statistics (kurtosis, median, and skewness), four GLCM (cluster-shade, cluster-prominence, energy, and sum-entropy), and one GLSZM size-zone-variability), and 47 filtered radiomic features remained and were included in the analysis. A brief description of the unfiltered and filtered features is shown in Supplementary Table S1.

### Data analysis

All analysis was performed using R software (version 3.2) with the “caret”^66^, Bioconductor “pROC” and “survcomp” packages^67^.

The association between radiomic features and binary semantic features was assessed using the area under the receiver operating curve (AUC). An AUC > 0.5 suggests direct proportionality between the radiomic and binary semantic features (i.e. a higher radiomic feature value corresponds to the presence of a binary semantic feature) and was defined as AUC_prop_. An AUC < 0.5 indicates inverse proportionality; that is, the presence of a binary semantic feature is associated with a low radiomic feature value. For AUC < 0.5, AUC_inv-prop_ was defined as 1-AUC. Both AUC_prop_ and AUC_inv-prop_ ranged from 0.50 to 1.00. We adapted the interpretation of the AUC from previous studies with 0.50 < AUCs ≤ 0.70, 0.70 < AUCs ≤ 0.90, and 0.90 < AUCs ≤ 1.00 to indicate weak, moderate, and excellent association^68, 69^. Noether’s test was used to determine the significance of the AUC from a random relationship (AUC = 0.5).

For the semantic features that were scored on the categorical scale, the strength and direction of their association with radiomic features were evaluated using the Spearman’s correlation coefficient (ρ). The cutoffs of |ρ| for weak, moderate, high, and excellent correlations was ≤0.50, 0.50 < |ρ| ≤ 0.70, 0.70 < |ρ| ≤ 0.90, and |ρ| > 0.90, respectively^70^. The Kruskal-Wallis test was used to assess the significance of the association.

All p-values were corrected for multiple hypothesis testing by adjusting the false discovery rate according to the Benjamini and Hochberg procedure^71^, where a q-value < 0.05 suggested statistical significance.

## Electronic supplementary material

Supplementary Information
