# Supplementary material for: Associations between radiologist-defined semantic and automatically computed radiomic features in non-small cell lung cancer
Source: Sci Rep. 2017 Jun 14;7:3519. doi: 10.1038/s41598-017-02425-5 (PMC5471260; doi:10.1038/s41598-017-02425-5)
Supplement: Supplementary file 1 — Supplementary Information [file 41598_2017_2425_MOESM1_ESM.pdf]

Associations between radiologist-defined semantic and automatically computed radiomic features in non-small cell lung cancer

Stephen SF Yip[1*], Ying Liu[2*], Chintan Parmar[1], Qian Li[2], Shichang Liu[2], Fangyuan Qu[2], Zhaoxiang Ye[2], Robert J. Gillies[3-4], and Hugo J.W.L. Aerts[1,5]

[1]Department of Radiation Oncology, Dana-Farber Cancer Institute, Brigham and Women's Hospital, and Harvard Medical School, Boston, MA 02115, U.S.A. [2]Department of Radiology, Tianjin Medical University Cancer Institute and Hospital, National Clinical Research Center, Key Laboratory of Cancer Prevention and Therapy, Tianjin, PR China [3]Department of Cancer Imaging and Metabolism, H. Lee Moffitt Cancer Center and Research Institute, Tampa, FL, U.S.A.; [4]Department of Radiology, H. Lee Moffitt Cancer Center and Research Institute, Tampa, FL., U.S.A. [5]Department of Radiology, Brigham and Women's Hospital and Harvard Medical School, Boston, MA 02115, U.S.A.

*These authors contribute equally

Corresponding author:

Stephen S.F. Yip, Ph.D.
Department of Radiation Oncology
Division of Medical Physics and Biophysics
Dana-Farber Cancer Institute and Harvard Medical School
Harvard Institutes of Medicine (HIM) building
3rd Floor – Suite 348
77 Avenue Louis Pasteur
Boston, MA 02115
312 804 1048 (cell)
Stephen_Yip@dfci.harvard.edu

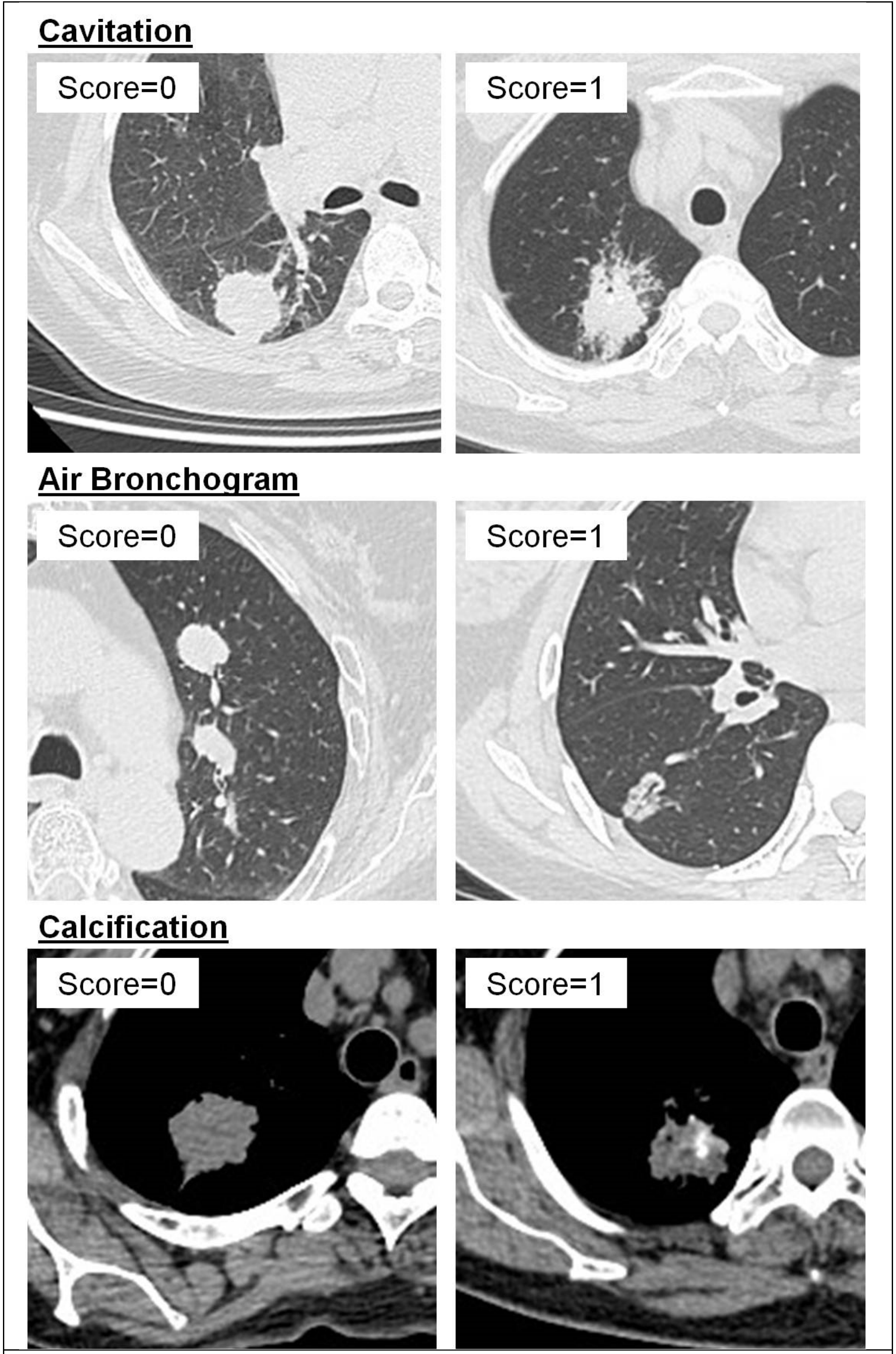

Supplementary Figure S1. Visual examples of binary semantic features

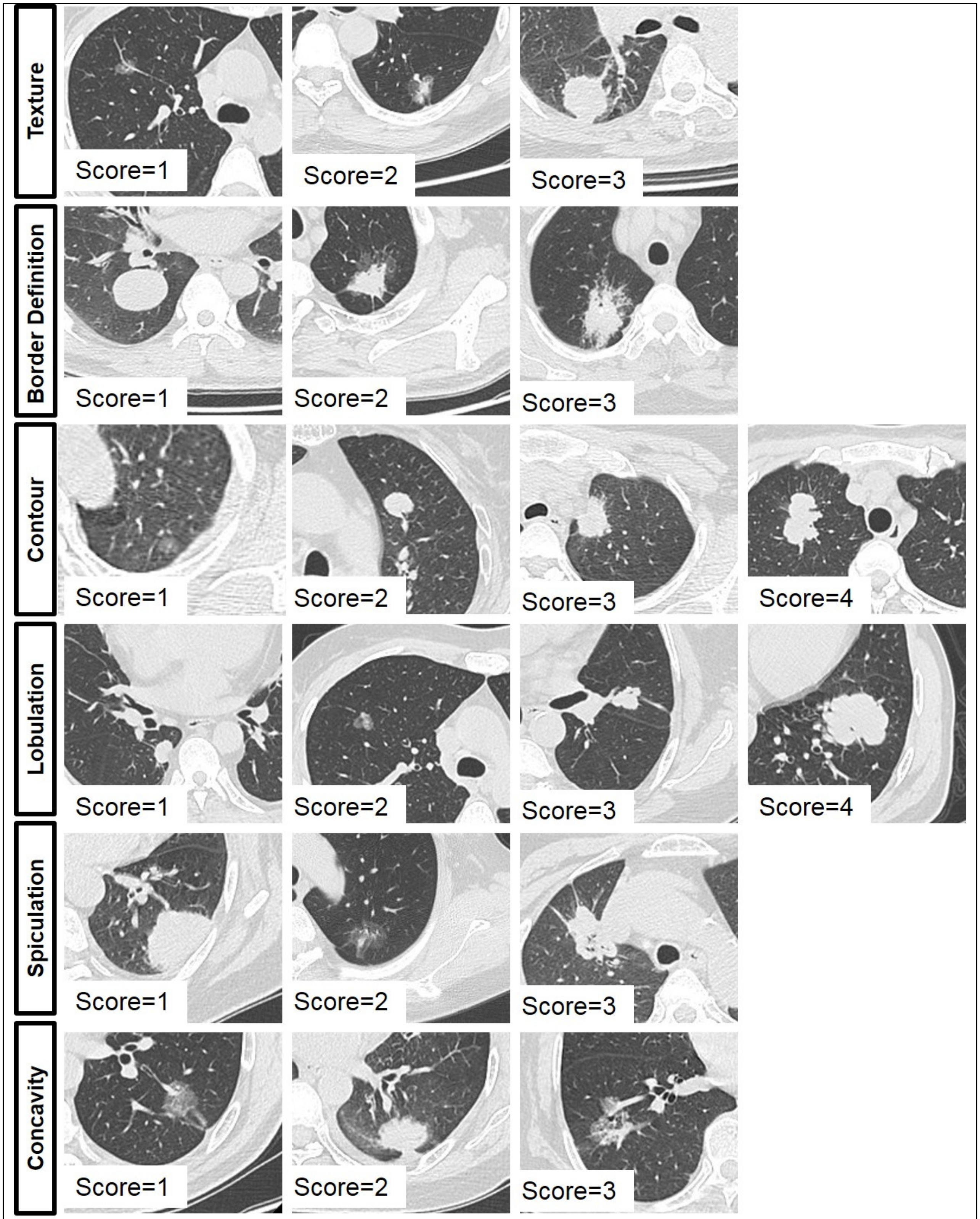

Supplementary Figure 2. Visual examples of categorical semantic features

Radiomic Feature Group	Radiomic feature	Description
**Shape**		
	Volume	Number of tumor voxels multiplied by the CT voxel volume
	Sphericity	Roundness of the tumor
**Statistics**		
	Median	Median value of the tumor Hounsfield unit (HU)
	Kurtosis	Measures the flatness of the tumor Hounsfield unit (HU) distribution (or how heavy-tailed or light tailed the distribution is) relative to a normal distribution
	Skewness	Measures the degree of asymmetry of the tumor HU distribution
**Gray level co-occurrence matrix (GLCM)**		
	Cluster Shade (clusShade)	Measures the asymmetric pattern within the tumor. High cluster shade value indicates great asymmetry
	Cluster Prominence (clusProm)	Measures the variation in Hounsfield unit (HU) within the tumor
	Energy	Measures tumor homogeneity
	Sum Entropy	Measures tumor heterogeneity
**Gray level size zone matrix (GLSZM)**		
	Size zone variability	Measures the similarity of the zones throughout the tumor; where a zone is a 3D volume with similar intensity value (HU).

Supplementary Table S1. Description of the radiomic features that were included in the analysis.

	Cavitation	Air.bronchogram	Calcification
GLCM_clusProm	0.76	0.64	0.45
GLCM_clusShade	0.27	0.40	0.53
GLCM_energy	0.39	0.41	0.53
GLCM_sumEntro	0.73	0.64	0.42
GLSZM_sizeZoneVariability	0.67	0.62	0.51
Shape_sphericity	0.41	0.40	0.51
Stats_kurtosis	0.40	0.46	0.68
Stats_median	0.38	0.41	0.64
Stats_skewness	0.47	0.47	0.37
Shape_volume	0.44	0.48	0.59
LoG_sigma_3_mm_3D_glcm_clusShade	0.29	0.39	0.64
LoG_sigma_3_mm_3D_glcm_clusTend	0.64	0.59	0.46
LoG_sigma_3_mm_3D_glcm_correl1	0.29	0.35	0.60
LoG_sigma_3_mm_3D_glcm_invDiffnorm	0.48	0.50	0.58
LoG_sigma_3_mm_3D_glcm_inverseVar	0.33	0.35	0.61
LoG_sigma_3_mm_3D_glcm_sumEntro	0.53	0.55	0.56
LoG_sigma_3_mm_3D_glszm_highIntensityLarteAreaEmp	0.53	0.54	0.51
LoG_sigma_3_mm_3D_glszm_largeAreaEmphasis	0.33	0.40	0.57
LoG_sigma_3_mm_3D_glszm_lowIntensitySmallAreaEmp	0.41	0.46	0.43
LoG_sigma_3_mm_3D_glszm_sizeZoneVariability	0.72	0.63	0.48
LoG_sigma_3_mm_3D_stats_mean	0.49	0.52	0.52
LoG_sigma_3_mm_3D_stats_uniformity	0.41	0.40	0.47
LoG_sigma_3_mm_3D_stats_uniformityPos	0.47	0.43	0.40
Wavelet_HHH_glcm_correl1	0.46	0.46	0.49
Wavelet_HHH_glcm_maxProb	0.33	0.39	0.48
Wavelet_HHH_glszm_lowIntensitySmallAreaEmp	0.35	0.38	0.48
Wavelet_HHH_glszm_sizeZoneVariability	0.65	0.61	0.51
Wavelet_HHH_stats_range	0.55	0.54	0.55
Wavelet_HHH_stats_std	0.65	0.60	0.46
Wavelet_HHL_glcm_inverseVar	0.25	0.34	0.59
Wavelet_HHL_stats_kurtosis	0.38	0.41	0.59
Wavelet_HHL_stats_min	0.48	0.55	0.45
Wavelet_HLH_glszm_lowIntensitySmallAreaEmp	0.32	0.42	0.48
Wavelet_HLH_stats_kurtosis	0.38	0.45	0.58
Wavelet_HLH_stats_min	0.48	0.49	0.45
Wavelet_HLL_stats_min	0.47	0.47	0.39
Wavelet_HLL_stats_range	0.49	0.51	0.62
Wavelet_HLL_stats_var	0.57	0.58	0.49
Wavelet_LHH_stats_kurtosis	0.39	0.43	0.61
Wavelet_LHH_stats_md	0.66	0.55	0.41
Wavelet_LHL_glszm_zonePercentage	0.73	0.64	0.38
Wavelet_LHL_stats_kurtosis	0.37	0.41	0.56
Wavelet_LHL_stats_mean	0.55	0.46	0.43
Wavelet_LHL_stats_var	0.60	0.52	0.45
Wavelet_LLH_glszm_lowIntensityEmphasis	0.34	0.37	0.47
Wavelet_LLH_glszm_sizeZoneVariability	0.71	0.62	0.51
Wavelet_LLH_stats_kurtosis	0.38	0.44	0.63
Wavelet_LLH_stats_max	0.51	0.45	0.57
Wavelet_LLH_stats_mean	0.59	0.49	0.41
Wavelet_LLL_glcm_correl1	0.65	0.59	0.49
Wavelet_LLL_glcm_maxProb	0.32	0.34	0.51
Wavelet_LLL_stats_kurtosis	0.39	0.42	0.58
Wavelet_LLL_stats_max	0.42	0.41	0.66
Wavelet_LLL_stats_min	0.31	0.40	0.57
Wavelet_LLL_stats_range	0.66	0.59	0.53
Wavelet_LLL_stats_totalenergy	0.62	0.56	0.50
Wavelet_LLL_stats_var	0.68	0.61	0.45

Supplementary Table S2. AUC values for the associations between the binary semantic and radiomic features

	Cavitation	Air.bronchogram	Calcification
GLCM_clusProm	1.00E-17	1.40E-04	0.2886
GLCM_clusShade	1.04E-13	1.04E-02	0.5903
GLCM_energy	3.88E-03	1.51E-02	0.5803
GLCM_sumEntro	1.37E-12	3.31E-05	0.1150
GLSZM_sizeZoneVariability	5.57E-07	1.20E-03	0.8207
Shape_sphericity	2.21E-02	1.03E-02	0.9100
Stats_kurtosis	6.50E-03	2.56E-01	0.0011
Stats_median	1.24E-03	8.83E-03	0.0053
Stats_skewness	4.23E-01	4.23E-01	0.0870
Shape_volume	1.79E-01	5.14E-01	0.1786
LoG_sigma_3_mm_3D_glcm_clusShade	2.54E-11	3.20E-03	0.0041
LoG_sigma_3_mm_3D_glcm_clusTend	1.26E-04	1.32E-02	0.4092
LoG_sigma_3_mm_3D_glcm_correl1	7.79E-10	7.19E-06	0.0469
LoG_sigma_3_mm_3D_glcm_invDiffnorm	8.50E-01	9.09E-01	0.4980
LoG_sigma_3_mm_3D_glcm_inverseVar	7.01E-07	1.98E-05	0.0261
LoG_sigma_3_mm_3D_glcm_sumEntro	4.50E-01	3.21E-01	0.3210
LoG_sigma_3_mm_3D_glszm_highIntensityLarteAreaEmp	5.40E-01	5.40E-01	0.9189
LoG_sigma_3_mm_3D_glszm_largeAreaEmphasis	3.25E-06	9.55E-03	0.2167
LoG_sigma_3_mm_3D_glszm_lowIntensitySmallAreaEmp	4.66E-02	2.76E-01	0.2756
LoG_sigma_3_mm_3D_glszm_sizeZoneVariability	6.99E-12	1.56E-04	0.7488
LoG_sigma_3_mm_3D_stats_mean	7.57E-01	7.57E-01	0.7571
LoG_sigma_3_mm_3D_stats_uniformity	1.69E-02	1.69E-02	0.5637
LoG_sigma_3_mm_3D_stats_uniformityPos	4.29E-01	6.94E-02	0.0694
Wavelet_HHH_glcm_correl1	4.16E-01	4.16E-01	0.8263
Wavelet_HHH_glcm_maxProb	1.91E-06	1.74E-03	0.7178
Wavelet_HHH_glszm_lowIntensitySmallAreaEmp	5.57E-05	9.10E-04	0.7682
Wavelet_HHH_glszm_sizeZoneVariability	6.03E-05	1.57E-03	0.8495
Wavelet_HHH_stats_range	3.13E-01	3.13E-01	0.3128
Wavelet_HHH_stats_std	3.51E-05	8.96E-03	0.4592
Wavelet_HHL_glcm_inverseVar	7.38E-15	2.55E-06	0.0766
Wavelet_HHL_stats_kurtosis	3.37E-03	1.37E-02	0.1093
Wavelet_HHL_stats_min	6.12E-01	3.80E-01	0.5945
Wavelet_HLH_glszm_lowIntensitySmallAreaEmp	1.07E-07	5.12E-02	0.6609
Wavelet_HLH_stats_kurtosis	1.59E-03	1.56E-01	0.1559
Wavelet_HLH_stats_min	6.79E-01	6.79E-01	0.6785
Wavelet_HLL_stats_min	4.05E-01	4.05E-01	0.0860
Wavelet_HLL_stats_range	8.44E-01	8.44E-01	0.0375
Wavelet_HLL_stats_var	9.18E-02	9.18E-02	0.9297
Wavelet_LHH_stats_kurtosis	8.85E-03	4.97E-02	0.0497
Wavelet_LHH_stats_md	8.02E-06	1.29E-01	0.1137
Wavelet_LHL_glszm_zonePercentage	2.71E-12	5.93E-05	0.0118
Wavelet_LHL_stats_kurtosis	8.98E-04	1.04E-02	0.2764
Wavelet_LHL_stats_mean	2.11E-01	2.37E-01	0.2108
Wavelet_LHL_stats_var	1.30E-02	5.81E-01	0.4448
Wavelet_LLH_glszm_lowIntensityEmphasis	2.37E-06	2.38E-04	0.5797
Wavelet_LLH_glszm_sizeZoneVariability	2.44E-10	8.77E-04	0.8284
Wavelet_LLH_stats_kurtosis	2.43E-03	8.17E-02	0.0119
Wavelet_LLH_stats_max	8.43E-01	1.97E-01	0.1965
Wavelet_LLH_stats_mean	4.65E-02	7.70E-01	0.1471
Wavelet_LLL_glcm_correl1	1.37E-05	2.26E-02	0.7602
Wavelet_LLL_glcm_maxProb	2.07E-07	2.14E-06	0.8333
Wavelet_LLL_stats_kurtosis	6.41E-03	2.85E-02	0.1740
Wavelet_LLL_stats_max	3.21E-02	1.27E-02	0.0005
Wavelet_LLL_stats_min	1.79E-08	8.26E-03	0.1246
Wavelet_LLL_stats_range	6.91E-06	1.86E-02	0.4979
Wavelet_LLL_stats_totalenergy	2.33E-03	1.78E-01	0.9798
Wavelet_LLL_stats_var	2.08E-07	2.50E-03	0.3554

Supplementary Table S3. False positive rate corrected p-value (q-value). q-value<0.05 indicates that AUC significantly different from random (AUC=0.50)

	Texture	Border.definition	Contour	Lobulation	Spiculation
GLCM_clusProm	-0.56	0.32	0.23	-0.08	-0.03
GLCM_clusShade	0.24	-0.33	-0.26	-0.03	-0.02
GLCM_energy	0.16	-0.28	-0.30	-0.14	-0.06
GLCM_sumEntro	-0.45	0.34	0.30	0.03	0.00
GLSZM_sizeZoneVariability	-0.35	0.31	0.29	0.09	0.05
Shape_sphericity	0.07	-0.42	-0.57	-0.32	-0.20
Stats_kurtosis	0.54	-0.11	0.02	0.28	0.15
Stats_median	0.65	-0.20	-0.10	0.22	0.19
Stats_skewness	-0.29	-0.07	-0.12	-0.16	-0.14
Shape_volume	0.23	0.09	0.25	0.38	0.11
LoG_sigma_3_mm_3D_glcm_clusShade	0.26	-0.23	-0.05	0.16	0.02
LoG_sigma_3_mm_3D_glcm_clusTend	-0.37	0.24	0.23	0.10	-0.01
LoG_sigma_3_mm_3D_glcm_correl1	0.52	-0.16	0.01	0.31	0.10
LoG_sigma_3_mm_3D_glcm_invDiffnorm	0.34	0.08	0.17	0.30	0.10
LoG_sigma_3_mm_3D_glcm_inverseVar	0.56	-0.17	-0.07	0.16	0.04
LoG_sigma_3_mm_3D_glcm_sumEntro	0.03	0.21	0.36	0.37	0.16
LoG_sigma_3_mm_3D_glszm_highIntensityLarteAreaEmp	0.14	0.14	0.19	0.28	0.04
LoG_sigma_3_mm_3D_glszm_largeAreaEmphasis	0.48	-0.13	0.00	0.28	0.07
LoG_sigma_3_mm_3D_glszm_lowIntensitySmallAreaEmp	0.00	-0.19	-0.25	-0.22	-0.12
LoG_sigma_3_mm_3D_glszm_sizeZoneVariability	-0.38	0.35	0.27	0.06	0.03
LoG_sigma_3_mm_3D_stats_mean	0.13	-0.09	-0.06	-0.06	0.08
LoG_sigma_3_mm_3D_stats_uniformity	0.21	-0.26	-0.34	-0.27	-0.11
LoG_sigma_3_mm_3D_stats_uniformityPos	-0.03	-0.17	-0.29	-0.34	-0.21
Wavelet_HHH_glcm_correl1	-0.01	-0.01	-0.02	-0.01	0.07
Wavelet_HHH_glcm_maxProb	0.16	-0.27	-0.32	-0.13	-0.07
Wavelet_HHH_glszm_lowIntensitySmallAreaEmp	0.23	-0.27	-0.28	-0.11	-0.02
Wavelet_HHH_glszm_sizeZoneVariability	-0.20	0.29	0.31	0.14	0.04
Wavelet_HHH_stats_range	0.00	0.25	0.29	0.26	0.19
Wavelet_HHH_stats_std	-0.25	0.30	0.19	-0.02	0.15
Wavelet_HHL_glcm_inverseVar	0.51	-0.24	-0.11	0.19	0.11
Wavelet_HHL_stats_kurtosis	0.29	-0.06	0.06	0.26	0.14
Wavelet_HHL_stats_min	-0.13	-0.16	-0.26	-0.30	-0.17
Wavelet_HLH_glszm_lowIntensitySmallAreaEmp	0.18	-0.23	-0.25	-0.14	-0.15
Wavelet_HLH_stats_kurtosis	0.25	-0.01	0.08	0.27	0.11
Wavelet_HLH_stats_min	-0.09	-0.22	-0.26	-0.28	-0.25
Wavelet_HLL_stats_min	0.04	-0.16	-0.28	-0.19	-0.12
Wavelet_HLL_stats_range	0.14	0.14	0.29	0.32	0.16
Wavelet_HLL_stats_var	-0.17	0.06	0.07	-0.04	0.04
Wavelet_LHH_stats_kurtosis	0.24	-0.07	0.03	0.23	0.02
Wavelet_LHH_stats_md	-0.26	0.21	0.11	-0.11	0.04
Wavelet_LHL_glszm_zonePercentage	-0.54	0.27	0.08	-0.26	-0.06
Wavelet_LHL_stats_kurtosis	0.37	-0.18	0.02	0.28	0.04
Wavelet_LHL_stats_mean	-0.20	0.04	0.04	0.05	-0.05
Wavelet_LHL_stats_var	-0.09	0.05	0.03	-0.04	-0.05
Wavelet_LLH_glszm_lowIntensityEmphasis	0.23	-0.24	-0.27	-0.12	-0.01
Wavelet_LLH_glszm_sizeZoneVariability	-0.31	0.34	0.32	0.11	0.07
Wavelet_LLH_stats_kurtosis	0.35	-0.10	0.07	0.30	0.03
Wavelet_LLH_stats_max	0.20	0.01	0.15	0.27	0.00
Wavelet_LLH_stats_mean	-0.27	0.09	0.02	0.05	-0.17
Wavelet_LLL_glcm_correl1	-0.37	0.35	0.31	0.03	0.05
Wavelet_LLL_glcm_maxProb	0.22	-0.31	-0.32	-0.05	-0.08
Wavelet_LLL_stats_kurtosis	0.37	-0.09	-0.01	0.29	0.01
Wavelet_LLL_stats_max	0.47	-0.02	0.10	0.26	0.14
Wavelet_LLL_stats_min	0.59	-0.21	-0.22	0.02	0.08
Wavelet_LLL_stats_range	-0.25	0.20	0.29	0.17	0.02
Wavelet_LLL_stats_totalenergy	-0.42	0.25	0.31	0.16	-0.03
Wavelet_LLL_stats_var	-0.44	0.22	0.17	-0.11	0.01

Supplementary Table S4. Associations between the categorical semantic and radiomic features assessed with Spearman coefficient

	Texture	Border.definition	Contour	Lobulation	Spiculation
GLCM_clusProm	4.01E-17	1.23E-05	1.93E-03	4.56E-01	0.7586
GLCM_clusShade	1.50E-06	6.61E-06	4.35E-04	3.58E-01	0.9482
GLCM_energy	1.19E-04	1.47E-04	1.39E-04	9.34E-02	0.1292
GLCM_sumEntro	5.50E-15	4.84E-06	1.83E-04	2.66E-01	0.9996
GLSZM_sizeZoneVariability	4.14E-08	1.14E-05	2.23E-04	4.10E-01	0.5906
Shape_sphericity	1.24E-02	7.67E-09	3.36E-16	2.45E-05	0.0127
Stats_kurtosis	9.43E-16	1.69E-01	9.60E-02	1.33E-04	0.0102
Stats_median	7.50E-23	1.03E-02	7.97E-02	2.72E-03	0.0116
Stats_skewness	7.72E-06	5.54E-01	1.87E-01	1.12E-01	0.0535
Shape_volume	3.68E-04	5.80E-02	7.29E-04	7.48E-07	0.0005
LoG_sigma_3_mm_3D_glcm_clusShade	4.53E-05	1.24E-03	1.55E-01	7.38E-03	0.1108
LoG_sigma_3_mm_3D_glcm_clusTend	4.14E-08	6.21E-04	3.06E-03	3.58E-01	0.9244
LoG_sigma_3_mm_3D_glcm_correl1	5.81E-15	2.54E-02	1.55E-01	9.72E-06	0.0102
LoG_sigma_3_mm_3D_glcm_invDiffnorm	2.02E-07	3.37E-01	9.86E-03	7.96E-05	0.0014
LoG_sigma_3_mm_3D_glcm_inverseVar	3.99E-17	3.50E-02	1.10E-01	4.61E-02	0.1056
LoG_sigma_3_mm_3D_glcm_sumEntro	4.34E-02	3.41E-03	1.07E-05	3.95E-06	0.0056
LoG_sigma_3_mm_3D_glszm_highIntensityLarteAreaEmp	3.29E-03	4.67E-02	1.44E-02	5.51E-04	0.0123
LoG_sigma_3_mm_3D_glszm_largeAreaEmphasis	3.56E-13	8.73E-02	1.74E-01	2.86E-04	0.0061
LoG_sigma_3_mm_3D_glszm_lowIntensitySmallAreaEmp	1.23E-02	9.97E-03	4.35E-04	1.34E-02	0.1572
LoG_sigma_3_mm_3D_glszm_sizeZoneVariability	1.55E-09	7.82E-07	5.40E-04	4.32E-01	0.4105
LoG_sigma_3_mm_3D_stats_mean	1.12E-01	1.14E-01	6.67E-01	7.38E-03	0.0078
LoG_sigma_3_mm_3D_stats_uniformity	3.45E-04	2.66E-04	1.91E-05	6.33E-04	0.0491
LoG_sigma_3_mm_3D_stats_uniformityPos	1.17E-01	2.71E-02	2.25E-04	9.72E-06	0.0073
Wavelet_HHH_glcm_correl1	9.44E-01	2.04E-02	3.02E-01	1.03E-01	0.5116
Wavelet_HHH_glcm_maxProb	3.34E-04	2.66E-04	1.01E-04	1.90E-01	0.5906
Wavelet_HHH_glszm_lowIntensitySmallAreaEmp	1.58E-04	1.95E-04	2.95E-04	3.58E-01	0.5167
Wavelet_HHH_glszm_sizeZoneVariability	9.61E-04	4.68E-05	1.02E-04	1.90E-01	0.5700
Wavelet_HHH_stats_range	1.63E-01	5.24E-05	1.83E-04	1.20E-03	0.0005
Wavelet_HHH_stats_std	4.34E-04	5.28E-05	8.68E-04	3.58E-01	0.0405
Wavelet_HHL_glcm_inverseVar	2.65E-14	1.24E-03	1.00E-01	2.29E-02	0.0241
Wavelet_HHL_stats_kurtosis	1.95E-05	1.32E-01	5.77E-01	5.96E-04	0.0011
Wavelet_HHL_stats_min	4.74E-02	2.66E-03	1.58E-03	1.95E-04	0.0045
Wavelet_HLH_glszm_lowIntensitySmallAreaEmp	3.34E-04	1.64E-03	1.76E-03	1.90E-01	0.0434
Wavelet_HLH_stats_kurtosis	4.23E-04	5.12E-02	3.28E-01	3.57E-04	0.0013
Wavelet_HLH_stats_min	4.67E-02	4.07E-04	4.35E-04	5.96E-04	0.0004
Wavelet_HLL_stats_min	6.14E-01	3.55E-02	3.58E-04	1.11E-02	0.0513
Wavelet_HLL_stats_range	3.25E-02	2.02E-02	3.48E-04	5.94E-05	0.0102
Wavelet_HLL_stats_var	2.66E-02	6.14E-01	1.52E-01	2.06E-01	0.1832
Wavelet_LHH_stats_kurtosis	8.95E-04	3.87E-01	2.76E-01	1.54E-04	0.0004
Wavelet_LHH_stats_md	2.52E-04	5.23E-03	3.64E-03	1.90E-01	0.0405
Wavelet_LHL_glszm_zonePercentage	6.81E-16	2.01E-04	1.10E-01	8.05E-04	0.0266
Wavelet_LHL_stats_kurtosis	5.20E-08	9.83E-03	7.97E-02	5.94E-05	0.0071
Wavelet_LHL_stats_mean	1.47E-03	2.79E-01	8.07E-01	1.92E-04	0.0509
Wavelet_LHL_stats_var	2.42E-01	7.26E-01	1.00E-01	4.32E-01	0.0078
Wavelet_LLH_glszm_lowIntensityEmphasis	1.20E-04	7.32E-04	5.40E-04	3.58E-01	0.4546
Wavelet_LLH_glszm_sizeZoneVariability	5.96E-07	2.66E-06	6.48E-05	3.69E-01	0.4654
Wavelet_LLH_stats_kurtosis	2.63E-07	8.73E-02	1.71E-01	5.94E-05	0.0004
Wavelet_LLH_stats_max	2.66E-03	3.44E-01	1.71E-01	1.54E-04	0.0050
Wavelet_LLH_stats_mean	1.20E-04	4.55E-02	4.61E-01	2.46E-03	0.0011
Wavelet_LLL_glcm_correl1	5.22E-12	2.28E-06	1.02E-04	5.44E-01	0.7266
Wavelet_LLL_glcm_maxProb	2.32E-05	2.40E-05	5.06E-05	2.83E-01	0.5210
Wavelet_LLL_stats_kurtosis	6.90E-08	2.79E-01	6.63E-03	7.04E-05	0.0321
Wavelet_LLL_stats_max	5.21E-13	3.37E-01	3.62E-01	5.65E-04	0.0134
Wavelet_LLL_stats_min	1.80E-18	5.41E-03	7.08E-03	7.38E-01	0.4297
Wavelet_LLL_stats_range	9.41E-09	4.14E-03	1.83E-04	7.21E-02	0.9482
Wavelet_LLL_stats_totalenergy	2.60E-10	1.59E-04	1.01E-04	1.12E-01	0.2139
Wavelet_LLL_stats_var	2.65E-14	4.14E-03	8.10E-03	4.55E-02	0.0450

Supplementary Table S5. False positive rate corrected p-value (q-value) obtained from Kruskal-Wallis test. q-value<0.05 indicates significant association between semantic and radiomic features
